# Peritonitis from Injury—Recovery

**Published:** 1850-11

**Authors:** 


### PATHOLOGY AND PRACTICE OF MEDICINE.

*Peritonitis from Injury—Recovery.*—It is our pleasing duty to record a case of severe injury to the abdomen, followed by very acute peritonitis, where, by the agency of opium, a recovery was obtained, which, under the circumstances, might have hardly been expected. It is by watching cases of this nature that we become convinced of the inestimable value of opium, and it may safely be asserted that innumerable lives have been saved by this powerful therapeutic agent. Mr. G. Mitchinson, the dresser, was kind enough to furnish us with ample details touching this very instructive case, and we forthwith proceed to lay them before our readers.

The patient is a stout, healthy-looking little boy, five years and a-half old, who was brought to St. Bartholomew's Hospital on the 10th of July, and placed in Kenton Ward, under the care of Mr. Stanley. The poor little fellow had, a few moments before his admission, been knocked down by a cart, the wheel of which was distinctly seen by several persons to have passed over the abdomen across the umbilicus. Immediately after the accident, the boy is said to have got upon his feet, and to have run some paces; but he soon bent the body forward, and lay down.

When he was brought to the hospital, he kept tossing from side to side, and seemed to suffer excruciating pain in the abdomen. The respirations were forty in a minute, suppressed and very short; the pulse about 140, and small; but he was so restless that the arterial pulsations could hardly be counted. The child had not vomited or passed any blood per anum; he stated that he had emptied his bladder about half an hour before the accident, and had just had his dinner. This latter statement can hardly be correct, as it is not very likely, as the dresser observed, that a child in that walk of life should dine at six.

However this may be, the little patient was at once given seven minims and-a-half of tincture of opium, and warm stupes were carefully applied to the abdomen. These means having given no relief, in about an hour the same dose of laudanum was repeated; the general symptoms remained the same; there was no vomiting, and the child was quite conscious. The patient remained in the same state during the next hour, rolling about in great agony. Neither the chest nor abdomen appeared to move much during respiration, which was short, frequent, and *catching*: the face was pale, but the extremities warm. As he was now inclined to doze, no more opium was given, though the child did *not* fall asleep, and went on tossing about, and moaning and crying with the pain in the abdomen. The same dose of laudanum was therefore repeated, and two ounces of wine given besides. In the night he was very restless; about an hour after the last dose of opium the child went to sleep, but woke several times, and complained bitterly of pain in his belly.

The bowels were not open towards the morning, nor was there any urine passed; a little retching took place, and the patient expectorated a small quantity of dark-coloured blood. During the day sleep was obtained; the pupils were, however, contracted, the respiration irregular though more natural and nasal. The pulse improved somewhat in volume, (130, intermittent), and the patient expressed himself as feeling better. On the third day, no urine had been passed since admission; an elastic catheter was therefore introduced into the bladder, and about eight ounces of clear and apparently healthy urine drawn off. The catheter was secured in the uretha, and about one ounce of turbid urine gradually drained away, loaded with an abundance of white flocculi.

When the patient awoke, about noon of the third day, he complained of great pain in the abdomen, and the respiration again became short and hurried; the child was given seven minims and a-half of tincture of opium, and two ounces of port wine; the urine on examination was found loaded with an abundance of lithates. Some milk and arrowroot were now taken with but little appetite, and the dose of laudanum, together with the wine, was repeated, but the child went on complaining of as much pain in the abdomen as before. On the fourth day he was somewhat quieter, and dozed a little; the pain and restlessness, however, soon returned, and the former was constantly referred to the abdomen. Tincture of opium, seven minums and a-half, and two ounces of wine. Towards the evening of this day, the ingestion of the opium was followed by marked relief: in fact, the child generally fell asleep about half an hour after taking each dose, and on awaking expressed himself relieved, although the pain never *ceased*, and after remaining awake a short time, it became so urgent, that he was afraid even to cough. He had, however, a little sleep for about two hours, after which time he was very restless and in pain.

The bowels had not been moved since admission; there was no vomiting, and the urine dribbled through the catheter. The same dose of opium and wine was repeated, and the patient now expressed great anxiety to have his medicine administered. On the fourth day there was not much alteration, except a fit of coughing, which occasioned a great increase of pain; the opium was repeated. The pulse was now 120, regular, soft and slightly improved in volume; the tongue somewhat furred and dry; the bowels still confined; and the urine passing through the catheter. No pain in the chest. In the afternoon the child fell into a deep sleep, which lasted three hours; on awaking he expressed himself as feeling much better, though coughing still caused much pain in the umbilical region. The pulse improved in volume, and was soft and compressible; the puipls not being so contracted as heretofore. Opium and wine repeated.

On the fifth day the patient seemed free from, pain, and the countenance was somewhat more natural and less expressive of uneasiness; the fits of pain, however, soon returned; the face became again extremely anxious; the pulse harder, and the respiration hurried. The opium and wine were repeated three times in the day without subduing the pain, which was still much complained of. Bowels still confined. Thus the patient went on for the next three days improving a little at times, but ever being subject to severe attacks of abdominal pain; the bowels remained inactive until the tenth day after admission, when, after a good night, he awoke considerably better, did not take his usual dose of opium and had a copious alvine evacuation. The pulse fell to 74, was soft and compressible, and the abdomen began to bear moderate pressure. The next few days were marked by gradual improvement, the child seldom required medicine, and did not experience any pain but when he committed some dietetic indiscretion; and about twenty days after admission the little patient left the hospital, to all appearance perfectly well, and has since remained so.

This case beautifully illustrates the rule of practice which states that the great point in the treatment is to support the system under the intense irritation excited by the visceral inflammation, and that no substance is so well calculated to effect this as opium. We are likewise advised, in contusion of the abdomen to avoid purgatives and enemata; this precept was here applied with the utmost strictness, as no alvine evacuation took place for nine days. Many would have been tempted to add a little calomel to the opium; but when we recollect the intense inflammation which must have existed to give rise to such continued pain, we are driven to admit that the least excitement in the shape of peristaltic motion might have raised the inflammation to a fatal degree.

It would appear from this case, that it was a favorable circumstance that the wheel, instead of passing across the hypocondrium, pressed upon the umbilical region, for the liver and spleen offering in the first place greater resistance than the convolutions of intestines, are more likely to be lacerated, which circumstance is almost always followed by the death of the subject. It is interesting to observe what large quantities of opium are borne under a high state of irritation of the system; the patient, a boy five years and a half old, took about twenty-two minims of laudanum per diem for about ten days without any unpleasant symptoms, and even without an unusual amount of sleep. In witnessing such a case as the preceding, the question naturally arises, whether the inflammation of the peritonæum has left any traces in the abdominal cavity, and whether the probable fibrinous exudations will eventually be absorbed, or interfere with the regular action of the bowels. The answer probably lies between the two extremes. We cannot close the report of this valuable case without adverting to the close connexion between medicine and surgery which is here illustrated, and we concur in the received opinion that a man cannot be a good surgeon, unless he at the same time be a good physician.—*Lon. Lancet.*

